# Electricity consumption of Singaporean households reveals proactive community response to COVID-19 progression

**DOI:** 10.1073/pnas.2026596118

**Published:** 2021-08-18

**Authors:** Gururaghav Raman, Jimmy Chih-Hsien Peng

**Affiliations:** ^a^Department of Electrical and Computer Engineering, National University of Singapore, Singapore 117581

**Keywords:** COVID-19, residential electricity consumption, behavior, pandemic response

## Abstract

Understanding how populations’ daily behaviors change during the COVID-19 pandemic is critical to evaluating and adapting public health interventions. Here, we use residential electricity-consumption data to unravel behavioral changes within peoples’ homes in this period. Based on smart energy-meter data from 10,246 households in Singapore, we find strong positive correlations between the progression of the pandemic in the city-state and the residential electricity consumption. In particular, we find that the daily new COVID-19 cases constitute the most dominant influencing factor on the electricity demand in the early stages of the pandemic, before a lockdown. However, this influence wanes once the lockdown is implemented, signifying that residents have settled into their new lifestyles under lockdown. These observations point to a proactive response from Singaporean residents—who increasingly stayed in or performed more activities at home during the evenings, despite there being no government mandates—a finding that surprisingly extends across all demographics. Overall, our study enables policymakers to close the loop by utilizing residential electricity usage as a measure of community response during unprecedented and disruptive events, such as a pandemic.

## Significance

It is vital for policymakers to understand how people react during a pandemic. Here, we propose to use domestic electricity-consumption data, which arguably capture peoples’ daily behaviors accurately and dynamically. Considering the city-state of Singapore as a case study, we study over 10,200 individual households’ electricity-consumption patterns to uncover previously unknown behavioral trends during the COVID-19 pandemic. While providing implications for the design of public health interventions during this and other pandemics, our results imply a proactive response from the community, which is surprisingly consistent across all demographics. This cohesive response may have helped the city-state in effectively curtailing the disease, a learning that has direct implications on the pandemic response of other nations as well.

Mitigation of the coronavirus disease 2019 (COVID-19) pandemic hinges on effecting massive behavioral changes in individuals across the world, at least until pharmaceutical interventions are developed and made available at scale (1, 2). In this context, it is imperative to accurately assess populations’ responses during the pandemic, which enables policymakers to adjust their interventions—particularly during critical periods, such as the initial stages of its progression—adaptively as well as retrospectively (3–5). For instance, showing that people are actively modifying their daily routines—e.g., by increasingly working from home and avoiding venturing into public spaces—can inform authorities about the extent to which they follow through on recommendations from public health experts. The challenge, then, is to identify specific measurable indicators that can constantly and accurately capture such behavioral changes.

By reviewing the pertinent literature, we have identified the following indicators that are currently being used to study social behavioral changes during the COVID-19 pandemic. The first indicator comprises responses gathered from the population by means of surveys. Thereby, researchers have attempted to obtain an overview of public perceptions (e.g., refs. 6 and 7). But this approach has several disadvantages: 1) Self-reported responses could either be untrue or exhibit a skew toward ideal or expected behaviors, rather than reflecting the reality (e.g., respondents could report that they are concerned about the pandemic and are self-isolating, while in reality taking no such actions); and 2) surveys only present snapshots of the population’s behavior at a particular time. Therefore, it may be difficult to glean any meaningful trends, given the fast-changing environment. The second indicator encompasses anonymized data from mobile phones, including passive geolocation data collected by mobile phone operators and actively collected contact-tracing data through dedicated applications (apps) (8–15). By determining the time spent by people at their homes and outside (e.g., in workplaces, shops, etc.) and analyzing how these behaviors change over time, recent studies (4, 5, 16–22) have attempted to discern the social response and design targeted interventions. However, this approach suffers from limitations as well: 1) Contact-tracing apps may not be used by many phone users, especially at the early stages of the pandemic; 2) individuals could own more than one mobile phone, or multiple individuals may share a phone; and 3) demographic differences in phone usage exist, with groups such as children and the elderly potentially underrepresented. These factors could distort the outcomes of such studies.

Yet, while the above indicators attempt to gain insight into people’s daily behaviors during the pandemic, surprisingly, studies in this context to date have not considered another potential indicator: residential electricity consumption. These data are routinely collected through smart energy meters, available to policymakers in real time, and avoid all of the previously mentioned limitations. Importantly, the electricity consumption of a household truly represents the occupants’ evolving at-home behaviors during the pandemic. In other words, there are no concerns of inaccuracies due to self-reporting. Secondly, since the electricity consumption of all the homes in the community is metered, regardless of their demographics, using electricity data to assess the population’s behavior will result in a more representative assessment. With this in mind, we study the Singaporean context and analyze the electricity consumption of 10,246 households in the city-state from January to May 2020. By tracking how the households’ electricity demands change during this period, we ascertain links between their behaviors and publicly available information about the progression of the pandemic. Our study shows that a strong positive correlation exists between the household peak consumption and new reported COVID-19 cases and that there is a lagged effect by 1 d. While the Singaporean residential electricity consumption is typically influenced predominantly by the weather (23–25), we find that in the early stages of the pandemic, progression of the disease has the most influence. This influence diminishes progressively as the country transitions into a strict lockdown—termed as the “Circuit Breaker”—in early April 2020, when people were only allowed to leave their homes for essential activities, such as grocery shopping and exercise. Overall, our findings underscore the proactive response of Singaporean residents to the pandemic, implying that they took steps to protect themselves, even before a government-mandated lockdown. This way, our study differs from the literature analyzing social behavior during the pandemic—while previous studies (19–22) have focused only on behaviors after mobility restrictions were put in place by authorities, we also analyze the period prior to their enforcement, assessing whether people adopted voluntary risk-mitigation behaviors.

It should be noted that we are not the first to study how electricity consumption has been affected by the pandemic. A recent study by Ruan et al. (17) has similarly examined the correlations between COVID-19 progression and the electricity consumption in different cities across the United States. However, in contrast to our study, their analysis was performed by using the *aggregate* demand, which includes not only the residential sector, but also the commercial and industrial counterparts. As we show later on, such an aggregation obfuscates trends in the residential demand, which is arguably the most direct indicator of the peoples’ daily behaviors, especially in a period when people are increasingly staying at home. Other studies (e.g., refs. 26–32) have also focused on the overall power sector in different countries during the pandemic, showing declining demand as lockdowns are enforced and commercial and industrial activities wind down. While these studies discern the power industry’s response to the pandemic, they provide limited insight insofar as the objective is to adjust public health interventions by analyzing the behaviors of the general public.

## Results

**Fig. 1.**
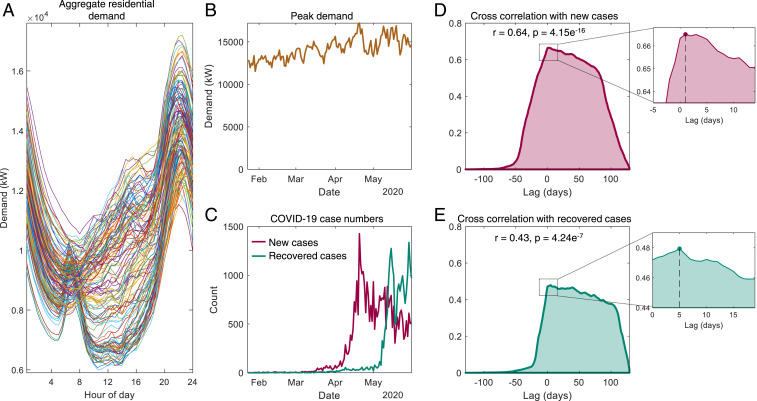
Relationship between COVID-19 case data and residential electricity consumption in Singapore. (*A*) Electricity-consumption profiles, aggregated for 10,246 households, for each day in the period of 23 January 2020 to 31 May 2020. (*B*) Daily peak values from *A*. (*C*) Daily new COVID-19 cases and recovered cases announced by the Ministry of Health Singapore. (*D*) Normalized cross-correlation plot between the peak aggregate demand and new COVID-19 cases. (*E*) Normalized cross-correlation plot between the peak aggregate demand and recovered cases. For *D* and *E*, the corresponding Pearson’s correlation coefficient and *P* value are indicated, and *Insets* present zoomed-in versions around the respective maximum cross-correlation values.

We obtained the electricity consumption of 10,246 households in Singapore from smart-meter data collected by their electricity service provider (*Materials and Methods*). With this, we assessed whether the residents proactively responded to public health authorities’ calls (33) to curtail the pandemic by avoiding crowded public places on a voluntary basis. Specifically, we studied if their evening-time electricity consumption increased, which would likely correlate with staying at home more during this time or shifting behaviors by doing more activities at home rather than outside. Note that while the entire populace may not have the flexibility to work from home before an official lockdown, everyone has available the option of avoiding crowded public places after work in the evening. To evaluate if this indeed happened in the initial stages of the pandemic in Singapore, we obtained the peak value of the aggregated residential consumption (which occurs in the evening; Fig. 1*A*) and studied if any relationships exist between the daily peak consumption and the progression of the pandemic. In particular, we used two metrics for the latter: the number of *daily new COVID-19 positive cases* and the number of *daily recovered cases* announced by the Ministry of Health through daily situation updates (34) and subsequently reported by the news media. It should be noted that these two variables constitute the only immediate information made available to the public that allow the people to assess the progression of the disease. We selected both of these data for our analysis due to their potential opposing influences on the society’s response—while the former may encourage people to be more cautious and avoid crowded public places, the latter may signal low disease severity to the public and encourage them to continue with business as usual. Fatality counts due to COVID-19 were not considered as a potential influencing factor, owing to the relatively low death toll in Singapore when compared globally and the fact that the first deaths happened only on 21 March 2020 (34) and cannot have had an effect on the population’s behaviors in January or February 2020.

Fig. 1*A* shows the daily aggregated demand of all the households for the period beginning on 23 January 2020—which is when the first COVID-19 positive case was detected in Singapore—and ending on 31 May 2020, until which the electricity demand data are available to us. Clearly, the daily peak always occurs in the evening from 8 PM to 11 PM; the corresponding peak values are obtained and plotted in Fig. 1*B*. From this figure, we observe that the peak demand continues to increase during this period. This trend would not be visible from analyzing the aggregate demand of the residential, industry, and commercial sectors. Indeed, such an aggregation would actually exhibit an opposite trend, given that residential demand only accounts for a small proportion of the total energy demand, about 14.9% in the Singaporean context (35). Therefore, the overall national demand reduced as the commercial and industrial activities ramped down during the pandemic (25). Similar declines in the overall demand were observed in other countries as well (17, 26–32). Toward our goal of assessing whether people respond to the progression of the disease, we now plot the COVID-19 case numbers for the same period in Fig. 1*C* and study the cross-correlation between the daily new cases and the peak demand (Fig. 1*D*) and that between the daily recovered cases and the peak demand (Fig. 1*E*). The corresponding Pearson’s coefficients r with the *P* value are also depicted in the figure. Here, while we observe statistically significant correlations (p≪0.05) between the peak aggregate demand and both the daily new and recovered COVID-19 cases, we find that the correlation between the latter pair is weaker. Further, from Fig. 1*D*, we find a maximum cross-correlation of 0.665 at a lag of 1 d, which suggests that the daily new cases have a 1-d-delayed association with the peak demand. Meanwhile, Fig. 1*E* shows a maximum cross-correlation of 0.479 at a lag of 5 d, implying that peak aggregate demand leads daily recovered cases by 5 d. To verify whether these correlations are spurious or represent a long-term relationship between the data, we tested for cointegration using the Engle–Granger cointegration test (36). For both new and recovered cases, the test indicates cointegration ($p=1e^{-3}\ll 0.05$ for both τ and z tests) with the peak demand values. These results suggest that there is indeed a link between the response of the society and progression of the disease. To examine this in more detail, we considered two distinct phases of the pandemic in Singapore: before the lockdown, or Circuit Breaker, which began on 7 April 2020; and during the Circuit Breaker. Analyzing the correlations during the two phases, we find statistically significant correlations for the former, but not the latter (*SI Appendix*, Note 1).

### Proactive Community Response before the Circuit Breaker.

An important question now arises about the above observations: Is it possible that the increase in the peak demand was not due to the response of Singaporeans toward COVID-19 progression, but was only caused by changes in the weather? We ask this because studies in the past have shown that the Singaporean electricity consumption mainly depends on the weather, with the demand generally increasing with the temperature (e.g., see refs. 23–25). Therefore, do the correlations shown in Fig. 1 *D* and *E* exist only because the weather becomes warmer, or is it also because of the social response to the pandemic?

**Fig. 2.**
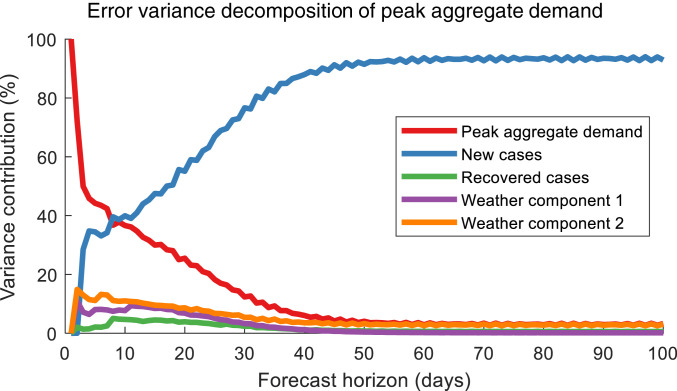
Results from the VECM for the peak electricity consumption of 10,246 households in Singapore. The figure shows the error variance decomposition of the influencing factors—daily new and recovered COVID-19 cases and two weather components—on the electricity consumption. The red plot corresponds to the portion of the forecast error variance in the peak aggregate demand that is unexplained by changes in the various influencing factors. Each of these variables experiences a one-SD shock at *x* axis equals zero. The VECM was trained with data from the period 23 January 2020 to 6 April 2020, which is the pre-Circuit Breaker period. Results demonstrate that the household electricity consumption is most influenced by changes in the daily new COVID-19 cases.

To answer this question, we constructed a vector error correction model (VECM) for the peak aggregate demand, while considering weather as a contributing factor. More specifically, five weather parameters were obtained and subsequently reduced to two principal components that explain more than 99.9% of the variance; see *Materials and Methods* for more details. In addition to these two weather principal components, the VECM was also fed the daily new and recovered COVID-19 cases as inputs. We then trained the model for the period beginning on 23 January 2020 until before 7 April 2020, when the government implemented the Circuit Breaker. Using this trained model, we performed forecast error variance decomposition to assess how changes in each factor contributed to the changes in the peak aggregate demand; in other words, we analyzed what the relative influence of each factor is on the households’ electricity consumption. These results are presented in Fig. 2. Note that the timeline for the forecast horizon is not to be confused with the actual time progression; rather, the analysis only shows the relative influence of the different influencing variables, given a trained VECM with no other exogenous shocks after the *x* axis equals zero. Three important observations can be gleaned from Fig. 2. First, it confirms that both COVID-19 progression and the weather influenced the electricity consumption. However, the most significant factor is the new COVID-19 cases—contributing over 93% of the variance—while the weather plays a relatively minor role, with the two components contributing about 3% combined to the variance. Second, although the government did not implement mobility restrictions prior to the Circuit Breaker, our results show that people proactively responded to the increasing new COVID-19 cases; the increasing electricity consumption suggests that people either stayed in to a greater extent or performed more activities at home rather than outside during the evenings. Finally, when comparing the roles played by the daily new COVID-19 cases and the daily recovered cases, we find that the influence of the latter is relatively negligible (less than 1%). Recall that we initially hypothesized that the population’s concerns may be alleviated by news of people recovering from their infections. Yet, we find that this is not the case, according to our VECM, suggesting that people responded more toward adverse news about the progression of the pandemic rather than patient recoveries.

### Impact of the Circuit Breaker.

**Fig. 3.**
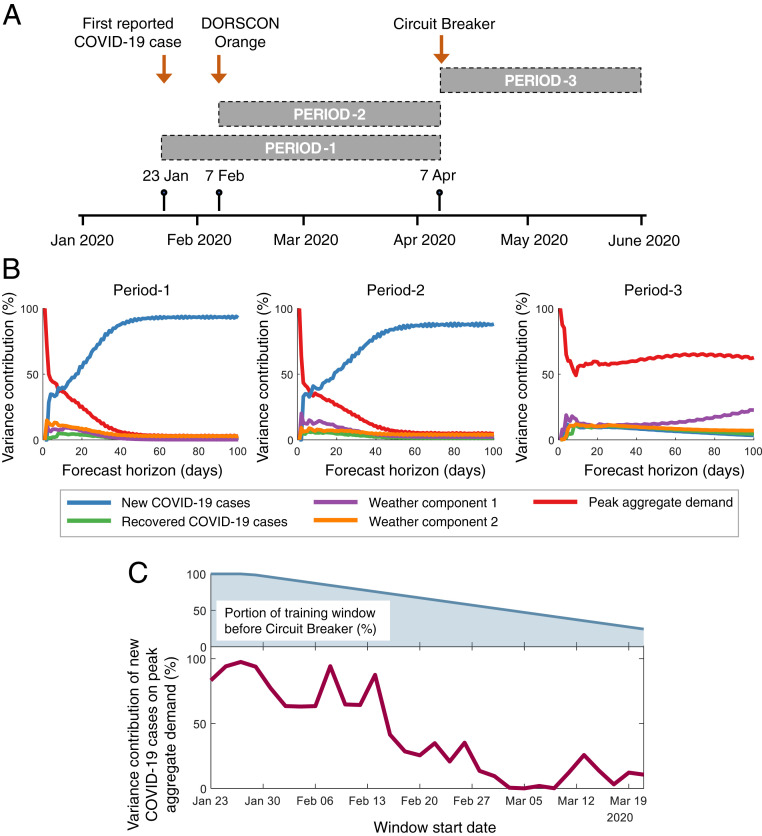
Impact of the Circuit Breaker on household electricity consumption. (*A*) We trained the VECM on three specific periods in 2020. Period-1 covers the interval from the reporting of the first COVID-19 positive case to the start of the Circuit Breaker. Period-2 also covers the pre-Circuit Breaker period, but begins after the government elevated the DORSCON to orange. Period-3 covers the interval after the Circuit Breaker begins. (*B*) Variance contributions of the different influencing factors on the peak residential consumption during the three periods indicated in *A*. (*C*) Illustrating how residents settle into their new lifestyles during the Circuit Breaker. Each point on the *x* axis indicates the starting date of a 10-wk window whose data are used to train the VECM. The figure depicts the forecast variance contribution of the new COVID-19 case numbers to the peak residential consumption for each time window, which moves forward in steps of 2 d each. It also depicts the portion of the training window that falls prior to the Circuit Breaker.

Having studied the pre-Circuit Breaker period, we now shift our attention to households’ electricity consumption as the country implements a full lockdown. We considered three specific time periods, as shown in Fig. 3*A* and explained below: 1) Period-1 corresponds to the pre-Circuit Breaker period, beginning on 23 January 2020, when the first positive COVID-19 case was reported, and ending on 6 April 2020. 2) Period-2 also covers the pre-Circuit Breaker period, beginning on 7 February 2020, when the Government of Singapore elevated the Disease Outbreak Response System Condition (DORSCON) to orange, indicating high disease severity and potential community transmission (37). 3) Period-3 covers the Circuit Breaker period, beginning on 7 April 2020 and ending on 31 May 2020, until which the residential demand data are available to us. For each period, we trained the VECM and plotted the extent to which each influencing factor contributes to the variance of the peak aggregate demand. This is shown in Fig. 3*B*.

Clearly, the influence of the new COVID-19 cases on the electricity consumption reduces as time progresses. Even during the pre-Circuit Breaker period—while it remains the most dominant factor—its influence on the peak demand during Period-2 falls to 89% from its original contribution of 93% in Period-1. Once the lockdown is implemented (i.e., during Period-3), however, its variance contribution is only 3.3%. As for the weather, we observe the opposite trend, with the combined influence of the two weather components increasing over time. While only contributing to 3.2% and 5.9% of the variance in the peak demand during Periods-1 and -2, respectively, their combined influence grows to 29.6% during the Circuit Breaker period. This results in weather becoming the dominant factor influencing the residential electricity consumption (excluding its own self-inertia). This is understandable, given the fact that the lockdown in Singapore was enforced strictly, and even first-time violators received substantial penalties (38); as such, the residents’ behaviors did not change significantly in this period due to the progression of the pandemic.

Until here, we have restricted our study to three specific time periods. Alternatively, we could employ a sliding time window and repeat the above analysis using VECMs trained for each of these windows. To this end, we consider a window 10 wk long, which iteratively moves forward in steps of 2 d. The results are presented in Fig. 3*C*, which depicts the variance contribution of the new COVID-19 cases toward the peak aggregate demand. The figure also depicts the proportion of the training window that falls prior to the Circuit Breaker—this value reduces as the window moves forward. Our results indicate that the influence of the new COVID-19 cases remains high, as long as the VECM training window lies outside the Circuit Breaker period. As the training window overlaps more and more with the Circuit Breaker period, the influence reduces. This implies that residents no longer responded to the progression of the pandemic by changing their behaviors and/or had grown accustomed to their new lifestyles under lockdown.

### Influence of Demographics.

**Fig. 4.**
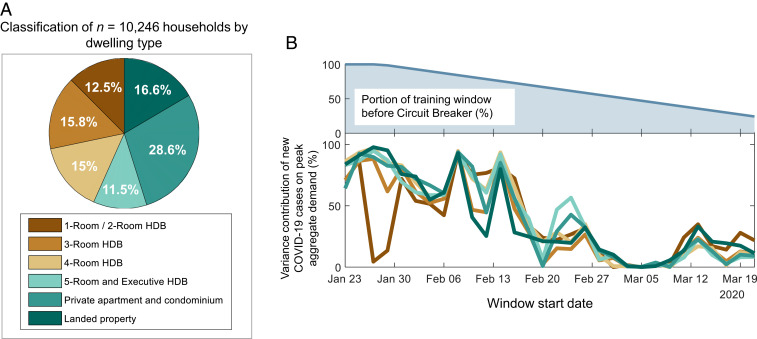
Influence of demographics on Singaporean residents’ response to the pandemic. (*A*) Classification of 10,246 Singaporean households by dwelling type. (*B*) The same as Fig. 3*C*, but for the aggregate demand of households belonging to different dwelling types.

Social response during the pandemic may be very different for different sections of the population, as evidenced by several recent studies (e.g., see refs. 20–22). To understand if demographic factors played a role in determining peoples’ response in the Singaporean context, we classified the 10,246 households into six different dwelling types: one-room/two-room HDB, three-room HDB, four-room HDB, five-room/executive HDB, private apartment/condominium, and landed property (*Materials and Methods*). (HDB here refers to residential apartments constructed by the Housing and Development Board, Singapore.) These dwelling types exhibit clear disparities in their family composition, average number of residents per household, and average income, with larger units more likely to be family homes with more residents and higher incomes (*SI Appendix*, Note 2). Results of the classification are summarized in Fig. 4*A*. For each dwelling type, we aggregated the demands of all the households belonging to that type. Subsequently, we employed the VECM that was described previously, but now train six different instances—each instance was trained with the peak aggregate demand of the corresponding dwelling type, along with the weather components. Fig. 4*B* presents the error variance decomposition results and shows that there are no significant differences in the reactions of the households based on the dwelling type. For each dwelling type, the overarching trend is consistent—the peak value of the electricity demand depends more on the progression of the disease during the initial stages of the pandemic than later on during the Circuit Breaker period. Referring to Fig. 4*B*, we observe a drop in the plot corresponding to households living in one-room/two-room HDB apartments for the period 25 to 31 January 2020. The overlap of this drop with the Chinese New Year holidays from 25 to 27 January 2020 suggests that any variation in the peak aggregate demand for these households is owing to the holidays, rather than the progression of the disease. The observation that only one-room/two-room HDBs depict this trend may be due to a large number of such households being nonfamily (*SI Appendix*, Note 2), and the Singaporean culture calls for family reunions during this widely observed holiday (39). We also analyzed the cross-correlation between new and recovered COVID-19 numbers and the aggregate demand for each dwelling type, which again shows similar responses by households, regardless of their demographics (*SI Appendix*, Note 3).

## Discussion

Our study has several implications. To begin, while residential electricity-consumption data have traditionally been used only for billing and grid-planning purposes, we have demonstrated that these data can capture peoples’ at-home behaviors in real time during a pandemic, adding to the list of bespoke data that are available to researchers and policymakers for this purpose. As noted earlier, this approach brings under the umbrella of analysis more people, such as the young and the elderly, who have been traditionally underrepresented in mobility data from smartphones. Encouragingly, smart electricity-meter data are becoming available in an increasing number of cities and countries (40). Next, although ours is a retrospective study, the analyses can be performed in real time to inform cities’ public health policies while tackling future waves of this, and other, pandemics. We highlight four specific ways below. First, policymakers can estimate the overall proclivity of the populace toward embracing risk-reduction behaviors. If people respond proactively at the beginning of the pandemic, they are most likely to maintain a responsive attitude to future interventions as well. While public attitudes may change over the long-term, e.g., due to fatigue (41), nevertheless, our study can anchor the necessary level of effort put into related public health campaigns, particularly in the absence of other real-time feedback. Second, policymakers can anticipate the speed of response of the community to their interventions. Our analysis suggests that Singaporean households responded to news of new COVID-19 cases with a delay of about 1 to 3 d (see high cross-correlations in Fig. 1*D* for a lag of 1 to 3 d, peaking at 1 d). Therefore, if a specific intervention in the near future fails to produce an impact within this time frame, it may portend the need to revise its design and/or implementation. Third, our VECM suggests that people respond more to public health updates that focus on the extent of the disease spread during the pandemic, i.e., the number of newly infected patients, rather than the number of recoveries. This has implications in designing future pandemic updates to the public. As such, our study suggests that Singaporean authorities’ efforts at the beginning of the pandemic were indeed effective in persuading Singaporeans about the severity of the disease and the need to effect immediate behavioral changes to tackle its spread. Fourth, policymakers can gain crucial insight into whether populations belonging to specific demographics require additional interventions, especially by combining electricity-consumption data with the corresponding location information (though unavailable to us due to privacy reasons). If at any time we find inaction among certain demographics, additional resources could be allocated to these groups. In particular, this approach can be harnessed during the global vaccine roll-out that is underway at the time of writing of this article; if certain groups are unable to reduce exposure, even during evenings, they can be targeted in the vaccination program. Specific to the Singaporean populace, our study suggests that households of all dwelling types responded in a cohesive manner before the lockdown to reduce the risk of exposure, despite significant disparities in their family composition, household size, and income. Given that to protect oneself during an infectious disease outbreak is to protect the society at large, this broad response could have contributed to the effectiveness of Singapore’s response to COVID-19. This observation from the Singaporean context is seemingly in contrast with studies of other populations [e.g., in the United States (20–22)] that have reported that socio-economic disparities do differentiate populations’ behavior during lockdowns. While cultural differences may have been a factor, we speculate that the differences arise because the aforementioned studies assess peoples’ behaviors during the day, which are predominated by their working habits, whereas we only consider their behavior during the evening. However, further studies are required to draw a definite conclusion.

Finally, we discuss the caveats of this study. First, as is the case for all observational studies, there is always the fear that all pertinent explanatory variables may not have been considered while explaining the observed behavior. In choosing the explanatory variables for the electricity-consumption changes in our study, we rely on existing studies (23–25) that show that the weather is the predominant factor influencing Singaporean residential electricity consumption and consider whether public information about the worsening pandemic changed the consumption patterns. On a related note, our study has established that the explanatory variables are Granger causes of the electricity consumption (*Materials and Methods* and *SI Appendix*, Note 4), but this is distinct from structural causality (42). Although techniques such as instrumental variable (43) or matching (44) could potentially be used to establish structural causality, the present study does not lend itself to these alternatives for several reasons: 1) Every household is assumed to consume news about the pandemic at the same rate, i.e., they are all “treated”; and 2) even if this were not the case, the data requirements for such analyses would be prohibitive. Second, while our experiments show that new COVID-19 cases influenced the peak household electricity consumption, they do not specify the particular modifications in the residents’ behavior or the underlying intentions that resulted in these demand changes. Our interpretation is that these stem from the populace proactively staying in during the evening to a larger extent and/or performing more activities at home rather than outside, with the intention of reducing potential exposure to the disease. In any case, one can argue that any behavioral change in response to the progression of the pandemic and in the absence of government mandates qualifies as a proactive response.

## Materials and Methods

The electricity-consumption data used in this study were obtained with approval from the Energy Market Authority (EMA), Ministry of Trade and Industry Singapore; contact ema_enquiry@ema.gov.sg for access. The codes used for our analyses are available at ref. 45.

### Data Collection.

#### Electricity-consumption data.

We obtained the smart-meter data of 11,901 unique residential consumers, who are supplied electricity by SP Group (46), with the consent of the EMA, Ministry of Trade and Industry, Singapore (47). This dataset consists of the kilowatt-hour consumption of the anonymized individual households at a half-hourly resolution for the period 1 November 2019 to 31 May 2020. From this dataset, we discarded households with missing entries. As a result, we obtained the complete electricity-consumption data for 10,246 households over the 7-mo period. While the original dataset did not specify the class of the households, i.e., the dwelling type, we used the average monthly electricity-consumption statistics (48) from the EMA to classify the households into six categories: one-room/two-room HDB, three-room HDB, four-room HDB, five-room/executive HDB, private apartment/condominium, and landed property. In more detail, each consumer’s average consumption during the months of November 2019, December 2019, and January 2020 was determined, and three separate classifications were performed by assigning to each consumer the dwelling type with the nearest average consumption, as reported by the EMA. The final classification was obtained as the majority of the three previous classifications. However, if all three classifications happened to be distinct, the consumer was assigned the dwelling type based on their November 2019 consumption.

#### Weather data.

Since the Singaporean electricity consumption is predominantly influenced by the weather (23–25), we included weather as an influencing factor while analyzing the change in the residential demand using our VECM. The weather data used in this study were obtained from Meteoblue (49). In particular, we obtained five different weather parameters at an hourly resolution for the time period under consideration: temperature, relative humidity, total cloud cover, solar irradiation, and wind speed. The first two parameters are measured at 2 m above the ground, while the wind speed is measured 10 m above the ground. Since these five parameters are highly correlated with each other, they were converted into two principal components, which explain over 99.9% of the variance, which serves to reduce the number of dimensions of the data.

#### COVID-19 case data.

We obtained COVID-19 case numbers released by the Ministry of Health Singapore to the media (50). Specifically, these consisted of the new positive COVID-19 cases and new recovered number of patients every day from January to May 2020.

#### VECM.

VECMs are used to capture complex relationships between multiple time-series data (51). An extension of vector autoregression models (52), VECMs are used when the time series to be analyzed are cointegrated, which is indeed the case for our analysis. Cointegration between variables exists when they are driven by a common stochastic trend; in such cases, there exist one or more linear combination of these variables that is stationary. The number of such linear combinations is referred to as the number of cointegration relations and is a parameter of the VECM. Another key component of the VECM is the degree of the multivariate autoregressive polynomial composed of the first differences of the time series, p. Here, (p+1) is the order of the vector autoregression model representation of the VECM.

In our study, the MATLAB econometrics toolbox was used to implement the VECM (53). The inputs to the model were the following: peak aggregate electricity demand, daily new COVID-19 cases, daily new recovered cases, and the first two principal components obtained from the five weather variables. Here, while the initial weather data were at an hourly resolution (i.e., 24 data points per day), the two components were averaged over each day in order to obtain a single data point per day per component. We verified if the four potential explanatory variables—daily new and recovered COVID-19 cases and the two weather components—were Granger causes of the peak aggregate electricity demand; see *SI Appendix*, Note 4 for the test results confirming Granger causality. The following steps were performed to train a VECM with data for a specific time period. Each of the input series was differenced, and their stationarity was verified by using the Augmented Dickey–Fuller test (54). Next, the number of cointegrating relations between the set of time series was found by using the Johansen cointegration test (55). The VECM was then fit to the inputs by using maximum likelihood (52). For each set of inputs, we varied the model parameter p in [0,6] and chose the value of p that minimized the Akaike information criterion (56). Finally, with this model, we performed forecast error variance decomposition (51), considering a forecast horizon of 100 steps. The parameters of the optimal VECM used for different simulations in this study are presented in *SI Appendix*, Note 4.

## Supplementary Material

Supplementary File

## Data Availability

Code has been deposited in the Power Engineering Lab site (https://www.penglaboratory.com/code-singaporean-covid19-response; ref. 45).

## Change History

August 25, 2021: The Acknowledgements have been updated.
