# Supplementary material for: Electricity consumption of Singaporean households reveals proactive community response to COVID-19 progression
Source: Proc Natl Acad Sci U S A. 2021 Aug 18;118(34):e2026596118. doi: 10.1073/pnas.2026596118 (PMC8403933; doi:10.1073/pnas.2026596118)
Supplement: Supplementary File [file pnas.2026596118.sapp.pdf]

PNAS
www.pnas.org

Supplementary Information for

Electricity Consumption of Singaporean Households Reveals Proactive Community Response to COVID-19 Progression

Gururaghav Raman and Jimmy Chih-Hsien Peng

Jimmy Chih-Hsien Peng.
E-mail: jpeng@nus.edu.sg

This PDF file includes:

Figs. S1 to S2
Tables S1 to S4
References for SI reference citations

1. Note 1: Correlations between the peak aggregate demand and COVID-19 case numbers

This section presents the summary of correlations between the peak electricity consumption of 10,246 households in Singapore and COVID-19 case numbers before, and during the lockdown, i.e., Circuit Breaker. Both Pearson and Spearman's correlation coefficients are presented, along with the corresponding p-values.

Table S1. Correlation between the peak electricity consumption and daily COVID-19 cases before and during the Circuit Breaker.

Time Period	Pearson		Spearman	
	Correlation coefficient	p-value	Correlation coefficient	p-value
**Peak aggregate consumption and new cases:**				
Pre-Circuit Breaker (23 Jan 2020 – 6 April 2020)	0.5446	4.3867e-07	0.5864	3.2384e-08
During Circuit Breaker (7 April 2020 – 31 May 2020)	0.2234	0.1011	0.1649	0.2290
**Peak aggregate consumption and recovered cases:**				
Pre-Circuit Breaker (23 Jan 2020 – 6 April 2020)	0.4517	4.7433e-05	0.4164	2.0235e-04
During Circuit Breaker (7 April 2020 – 31 May 2020)	0.1417	0.3022	0.1438	0.2950

From Table S1, we observe statistically significant ($p << 0.05$) correlations between the peak residential electricity consumption and both new and recovered COVID-19 cases in the pre-Circuit Breaker period, but not during the Circuit Breaker ($p > 0.05$).

2. Note 2: Demographic data for Singaporean households

We classify the 10,246 consumers into six dwelling types using statistics from the Energy Market Authority (EMA) Singapore (1) and the procedure detailed in the Methods section of the main article. The results of the classification are presented in Fig. 4(a) in the main article.

Fig. S1 below presents the average monthly household income from work for the different dwelling types as reported by the Department of Statistics, Singapore (2). Also shown are data regarding the number of persons living on an average in each dwelling type (2). Furthermore, we obtained the latest available data (from 2018) regarding what percentage of residents in a particular dwelling type (only data for HDB apartments are available) are families or non-families (3); Table S2 presents these results. These data clearly illustrate the socio-economic disparities amongst the various dwelling types.

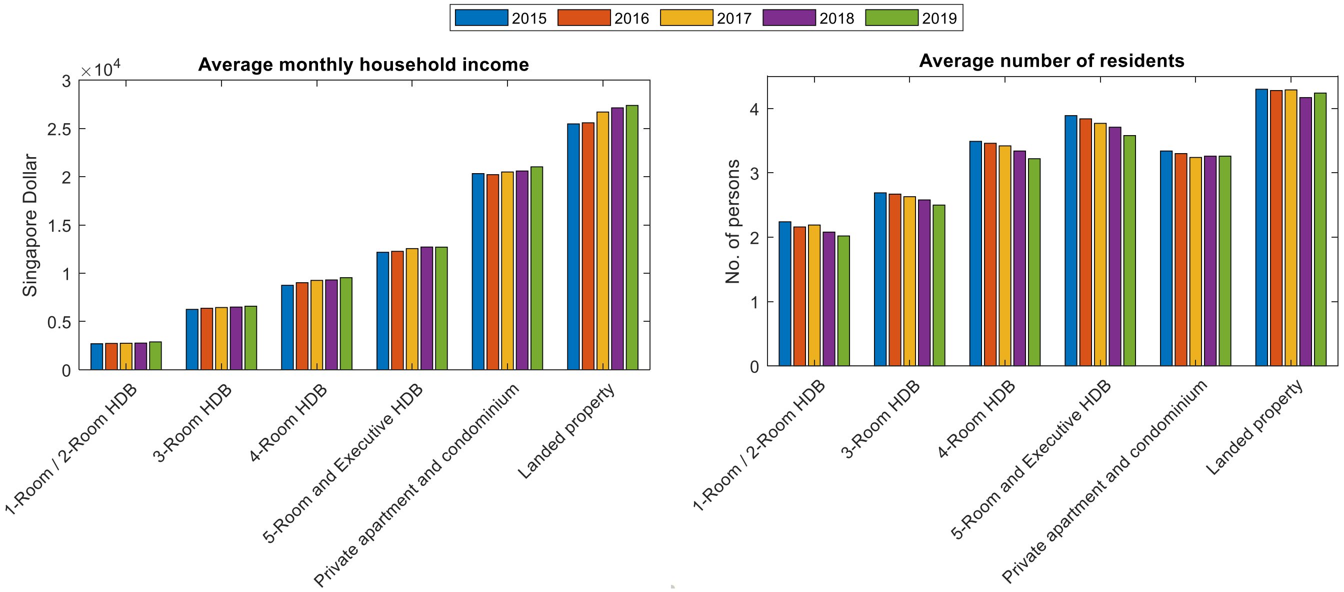

Fig. S1. Average monthly income from work and household size by dwelling type for Singaporean households in the period 2015–2019. [Data source: (2)]

Table S2. Family characteristics of HDB apartment residents by dwelling type in 2018. [Source for data and definitions: (3)]

Dwelling type	Type of family nucleus (%)			
	Nuclear family	Extended nuclear family	Multi-nuclear family	Non-family based households
1-Room HDB	49.3	2.1	— †	48.2
2-Room HDB	66.4	4.1	1.2	28.3
3-Room HDB	66.9	4.2	3.0	26.0
4-Room HDB	78.3	7.7	5.0	8.9
5-Room HDB	83.3	6.6	5.7	4.3
Executive HDB	80.2	9.1	7.2	3.6

† Values with high coefficient of variation were dropped in the original dataset and are not available.

Nuclear family refers to a married couple with/without children, or a family consisting of immediate related members.

Extended nuclear family refers to a nuclear family with one or more relatives.

Multi-nuclear family refers to a family with two or more nuclear families.

Non-family based households refer to one-person households, or those with unrelated persons staying together.

3. Note 3: Cross-correlation between the peak aggregate demand and COVID-19 case numbers for different dwelling types

For the households belonging to each dwelling type, we aggregate their electricity consumption and calculate the cross-correlation between the peak aggregate demand and daily COVID-19 case numbers. The results are presented in Fig. S2 below.

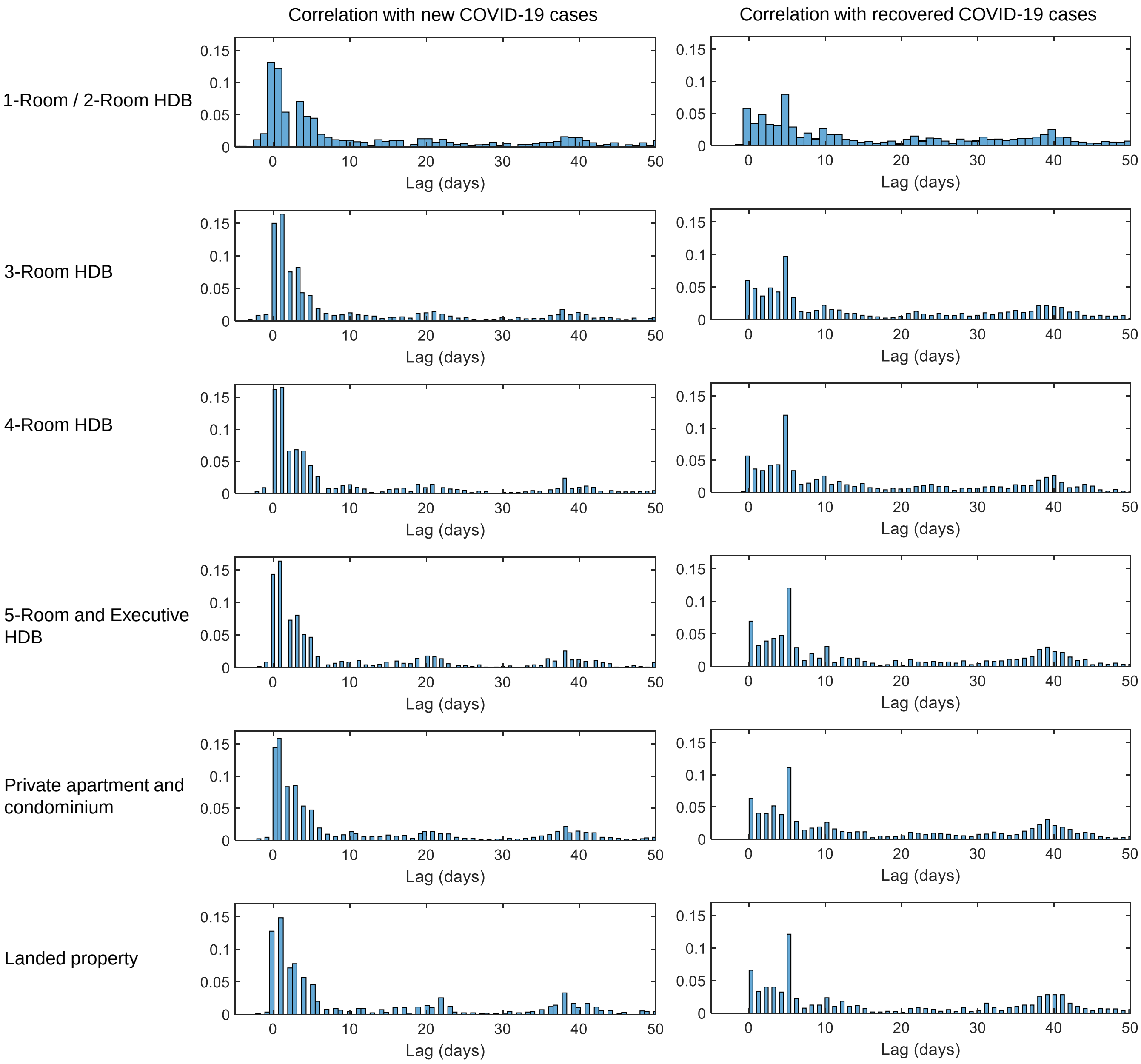

Fig. S2. Cross-correlation between the peak aggregate electricity consumption of households grouped by dwelling type and daily new/recovered COVID-19 cases for the period of 23 January to 31 May 2020.

From this figure, we observe no significant differences in the responses of the households belonging to different dwelling types.

4. Note 4: Vector error correction model parameters

We employ vector error correction models (VECMs) to capture relationships between the households' peak aggregate electricity consumption, daily new and recovered COVID-19 case numbers, and two weather components. As explained in the Methods section of the main article, the main parameter of the VECM is the degree of the multivariate autoregressive polynomial composed of the first differences of the time series, p, where $(p+1)$ is the order of the vector autoregression model representation of the VECM (4). Another parameter is the rank of the VECM, which refers to the number of cointegration relations in the input time series.

Table S3 presents the VECM parameters that minimize the Akaike information criterion (AIC) for the three time periods considered in Fig. 3 in the main article and listed below for convenience:

1. Period-1 corresponds to the pre-Circuit Breaker period, beginning on 23 January 2020 when the first positive COVID-19 case was reported in Singapore and ending on 6 April 2020;

2. Period-2 also covers the pre-Circuit Breaker period, beginning on 7 February 2020 when the Government of Singapore elevated the Disease Outbreak Response System Condition (DORSCON) to Orange—indicating high disease severity and potential community transmission—and ending on 6 April 2020;

3. Period-3 covers the Circuit Breaker period, beginning on 7 April 2020 and ending on 31 May 2020 until which the residential demand data is available to us.

Table S3. VECM parameters.

Period	Best p	Rank of VECM
1	6	3
2	6	3
3	6	2

Causality test. For each of the three time periods, we tested (5) whether the four potential explanatory variables—daily new and recovered COVID-19 case numbers and two weather components—Granger-cause the peak aggregate electricity consumption values. Granger causality is based on the idea that a cause always appears before the effect. Therefore, suppose a variable x causes a variable z, then, the former should improve the accuracy of predictions of the latter. For more explanation and mathematical definitions, see (4). The results of the causality tests are summarized in Table S4 while considering a significance level of 0.05.

Table S4. Results of the Granger causality tests.

Period	Granger causality	p-value
1	Yes	0.010
2	Yes	0.0286
3	Yes	$4.558e^{-10}$

For all of the three time periods, we find that the test rejects the null hypothesis that the explanatory variables do not Granger-cause the peak aggregate electricity consumption, with a p-value less than 0.05 in each case. In other words, the test confirms that the four explanatory variables Granger-cause the peak aggregate demand.

References

1. EMA Singapore, Average monthly household electricity consumption by dwelling type (https://www.ema.gov.sg/statistic.aspx?sta_sid=20140617E32XNb1d0Iqa) (2020).
2. Department of Statistics Singapore, Key household income trends, 2019 (https://www.singstat.gov.sg/-/media/files/publications/households/pp-s26.pdf) (2019).
3. Housing and development board, Public housing in Singapore: Residents' profile, housing satisfaction and preferences (https://www.hdb.gov.sg/-/media/HDBContent/Images/CDG/Library/Library/SHS-2018-Monograph-1---10-Feb-2021.pdf) (2021).
4. H Lütkepohl, *New introduction to multiple time series analysis.* (Springer Science & Business Media), (2005).
5. MathWorks, Block-wise Granger causality and block exogeneity tests (https://www.mathworks.com/help/econ/gctest.html) (2021).
